# Fluoroscopy‐Guided Localization and Retrieval of a Migrated Extrapelvic Intrauterine Device: A Case Report

**DOI:** 10.1155/crog/2546849

**Published:** 2026-08-12

**Authors:** Mohammad Haekal, Caroline Gladys Puspita, Tiara Sirinding Mangiwa, Mazkuroh Urfah, Gde Suardana

**Affiliations:** ^1^ Department of Obstetric and Gynecology, Harapan Kita National Women and Children Health Centre, Jakarta, Indonesia

**Keywords:** extrapelvic migration, fluoroscopy assisted laparoscopy, intrauterine device, uterine perforation

## Abstract

**Background:**

Intrauterine devices (IUDs) are widely used as a highly effective method of contraception. Although rare, uterine perforation is a serious complication, occurring in approximately 1 per 1000 insertions. This case underscores the value of laparoscopy in enabling accurate diagnosis and immediate removal of an occult extrauterine IUD migration. In addition, fluoroscopy guidance improves localization accuracy, facilitates precise retrieval, and may help prevent laparotomy.

**Case Presentation:**

This case presents a 25‐year‐old woman presented with progressive abdominal pain 1 year after IUD insertion. Physical examination revealed no involuntary guarding and the IUD strings were not visible. Transvaginal ultrasonography showed no evidence of a myometrial defect. An abdominal x‐ray with uterine marker demonstrated extrapelvic migration of the IUD. The patient subsequently underwent fluoroscopy‐assisted laparoscopic retrieval. Intraoperative findings revealed a uterine scar and dense adhesions between the IUD and the omentum.

**Conclusion:**

Early clinical suspicion and a structured imaging approach are essential for the diagnosis of extrauterine IUD migration. When pelvic ultrasonography is inconclusive, abdominal radiography with uterine markers can improve localization. Fluoroscopic guidance improves intraoperative localization and facilitates safe minimally invasive retrieval of extrapelvic IUD migration while minimizing surgical morbidity.

## 1. Background

Intrauterine contraceptive devices are widely used as highly effective, long‐term, and reversible methods of contraception [1]. Despite their safety profile, uterine perforation remains a rare but serious complication of insertion, occurring in approximately 1 per 1000 cases [2]. The extent of perforation ranges from partial embedment within the myometrium to complete migration into the peritoneal cavity. Gill reported an incidence of uterine perforation ranging from 0.2 to 3.6 per 1000 insertions [3].

Migrated intrauterine devices (IUDs) have been identified in various anatomical locations, including the omentum, pouch of Douglas, colonic lumen, broad ligament, abdominal cavity, small bowel serosa, mesentery, and ovary. Accurate preoperative localization is therefore essential to reduce patient morbidity [4]. This case highlights laparoscopy as an optimal approach for diagnosing and simultaneously removing a extrauterine migrated IUD, with fluoroscopic assistance serving as a useful adjunct for precise localization and prevention of conversion to laparotomy.

## 2. Case Presentation

**Figure 1:**
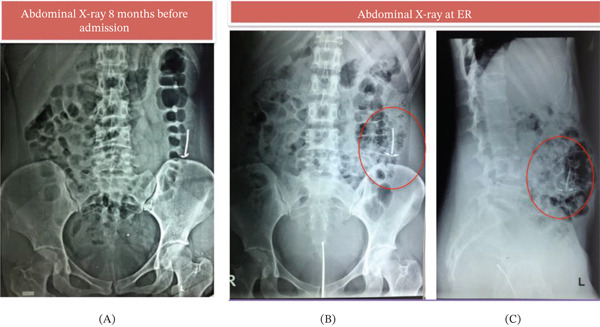
Plain abdominal x‐ray demonstrating the extrapelvic migration of the intrauterine device (IUD). (A) AP projection of abdominal radiography 8 months before the admission. (B) AP and (C) left lateral projection with the uterine marker at emergency room.

A 25‐year‐old woman presented to the emergency department with left‐sided abdominal pain (VAS score: 5) that had begun 2 h prior to admission. She had delivered her first child vaginally last year and elected to undergo IUD insertion 3 months after birth performed by the midwife. She experienced mild cramping immediately after the insertion and noticed recurrent abdominal pain over the following 2 months. She reported no urinary symptoms, bowel disturbances, or dyspareunia after the insertion. She consulted then to a physician, and extrapelvic IUD migration was suspected after a plain abdominal radiograph demonstrated an extrauterine position of the device (Figure 1A). IUD removal was recommended; however, she declined and opted for analgesic therapy alone.

On physical examination at the ER, there was no abdominal guarding, and the IUD strings were not visible. Transvaginal ultrasonography revealed no myometrial defects and no IUD within the uterine cavity. Subsequently, an abdominal x‐ray with a uterine marker was performed in anteroposterior (Figure 1B) and lateral views (Figure 1C). The device was visualized at the L4–L5 level in the anterior left hemiabdomen, located 16.6 cm from the uterine marker on the anteroposterior projection and 15.8 cm on the lateral view, consistent with extrauterine IUD migration.

**Figure 2:**
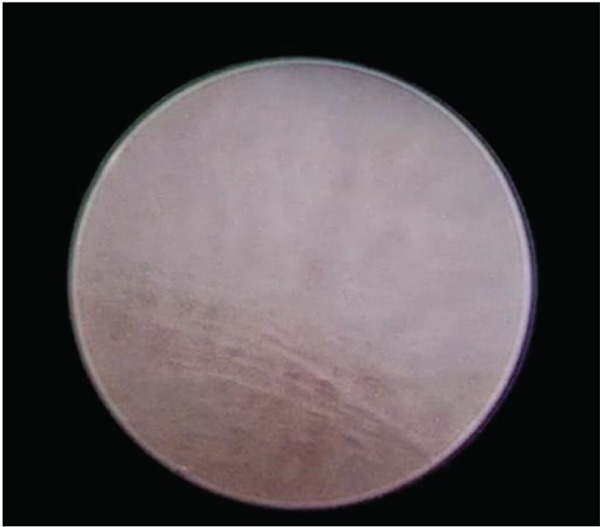
Normal uterine cavity.

On the following day, the patient provided informed consent to undergo surgical removal of the device. A combined hysteroscopic and laparoscopic approach was undertaken, with fluoroscopic guidance available to facilitate precise localization of the IUD. Hysteroscopy was conducted to assess the uterine cavity for residual defects and demonstrated a normal cavity without evidence of perforation or retained intrauterine pathology (Figure 2).

**Figure 3:**
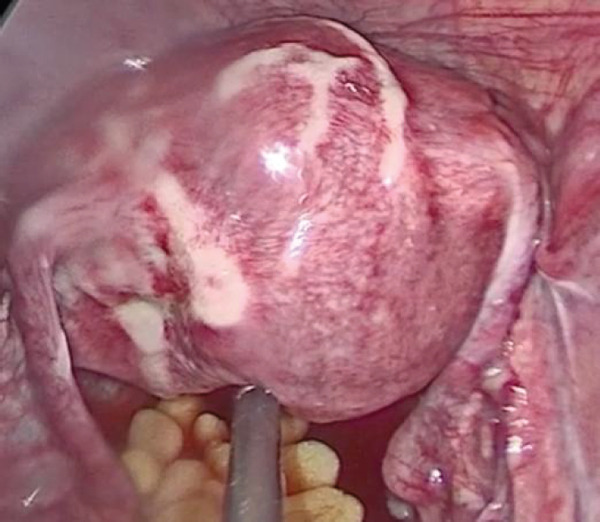
Uterine scar.

**Figure 4:**
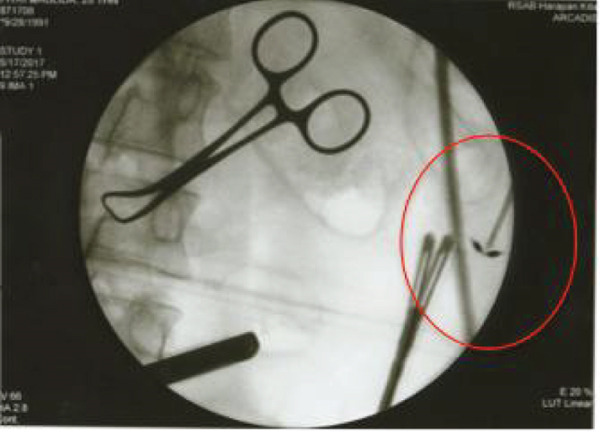
Fluoroscopy appearance of IUD.

**Figure 5:**
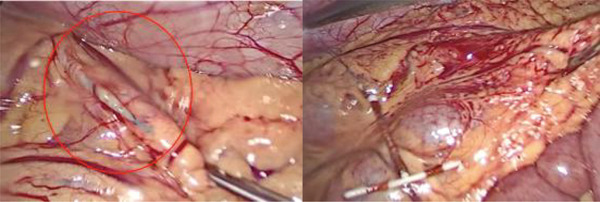
IUD embedded within omentum.

Laparoscopy was initiated using a single umbilical trocar to minimize surgical trauma. The procedure revealed uterine scarring in the left lateral and fundal regions (Figure 3); however, the IUD was not immediately visualized. Correlation of preoperative abdominal radiographs with intraoperative fluoroscopic imaging enabled accurate localization of the device (Figure 4). Fluoroscopy also helped avoid conversion to laparotomy. The IUD was ultimately identified in the left lateral abdomen, adjacent to the descending colon, and was noted to be densely embedded within the omentum (Figure 5). Due to omental adherence, two additional accessory trocars were placed to facilitate safe dissection. The IUD was successfully removed without complication. The postoperative course was uneventful, and the patient was discharged on the following day.

## 3. Discussion

IUD migration should be suspected in patients presenting with abdominal pain following insertion, absence of visible IUD strings on physical examination, failure to identify the device on pelvic ultrasonography, and abdominal radiographs indicating an extrauterine position [5]. In the present case, these clinical and imaging findings were concordant and supported the diagnosis. Kho and Chamsy reported that abdominal pain was present in 54% of patients with IUD migration, whereas 43% were asymptomatic, emphasizing the importance of imaging even in the absence of symptoms [4]. Therefore, systematic evaluation—including history taking, physical examination, and appropriate imaging—is essential for timely diagnosis.

Pelvic ultrasonography remains the first‐line imaging modality to assess IUD position and detect possible embedment or perforation. However, ultrasonography alone may be insufficient when the device is completely extrauterine [5]. In such cases, anteroposterior and lateral abdominal radiographs are necessary to confirm migration and assess the extent of displacement. The use of a uterine marker during radiography, as performed in this case, enhances spatial orientation, and facilitates accurate preoperative localization relative to surrounding anatomical structures, which is particularly useful for surgical planning.

Although uterine perforation occurring at the time of IUD insertion is the most widely recognized mechanism of device migration, alternative pathways have also been proposed. Primary perforation typically results from direct penetration of the uterine wall during insertion and is associated with factors such as postpartum insertion, lactation, uterine anomalies, and operator experience [6]. However, some cases of extrauterine IUDs are diagnosed months or even years after apparently uncomplicated insertion, suggesting that delayed secondary perforation may contribute to migration. This mechanism involves gradual erosion of the IUD through the myometrium driven by chronic inflammatory reactions, uterine contractions, or pressure necrosis exerted by the device on the uterine wall. Over time, the IUD may progressively traverse the myometrium and serosa, eventually entering the peritoneal cavity and becoming embedded in adjacent structures such as the omentum, bowel, or bladder [7]. The absence of immediate symptoms following insertion and the delayed presentation observed in many reported cases support the hypothesis that migration may occur as a dynamic process rather than solely as an insertion‐related event. Recognition of these alternative mechanisms is clinically important because patients with delayed perforation may remain asymptomatic for prolonged periods, leading to delayed diagnosis and an increased risk of adhesion formation and involvement of surrounding organs. Consequently, a missing IUD string should prompt thorough evaluation regardless of the time elapsed since insertion [6, 7]. In the present case, both mechanisms may have played a role in the migration of the IUD. Uterine scarring identified in the left lateral and fundal regions during laparoscopy is consistent with delayed secondary perforation, whereas the occurrence of pelvic pain shortly after insertion raises the possibility of insertion‐related perforation. Therefore, the clinical and intraoperative findings suggest that either mechanism, or a combination of both, may have contributed to the extrauterine displacement of the device.

The World Health Organization (WHO) recommends prompt removal of a dislocated IUD once uterine perforation and extrauterine displacement of an IUD have been confirmed to prevent potential long‐term complications [8]. Delayed intervention increases the risk of adhesion formation and secondary complications. Even in asymptomatic patients, retained devices may lead to long‐term complications such as adhesion formation, chronic pelvic pain, abscess formation, bowel obstruction, bowel perforation, fistula formation, and involvement of adjacent organs including the bladder and omentum [5, 7]. The interval between IUD migration and surgical retrieval may significantly affect procedural complexity, morbidity, and clinical outcomes. Prolonged retention of a migrated IUD can lead to secondary complications resulting from chronic inflammation and foreign body reactions. For instance, one case report described an IUD retained within the urinary bladder that became encrusted and served as a bladder stone formation, requiring lithotripsy before successful cystoscopy extraction of the device [9]. Reported sites of intra‐abdominal IUD migration include the omentum, Douglas pouch, colonic lumen, myometrium, broad ligament, and small bowel serosa, with rarer involvement of the bladder, appendix, ovary, and retroperitoneum [4]. Minimally invasive surgery, particularly laparoscopy, is considered the preferred approach when feasible because it allows effective retrieval with lower postoperative morbidity than laparotomy [2, 3, 7]. Although some asymptomatic patients may remain complication‐free for prolonged periods, the unpredictable nature of extrauterine IUD migration and the potential for delayed organ injury support the current recommendation for surgical removal whenever technically possible [7]. In this case, the IUD was embedded within the omentum, consistent with previous reports and supporting the hypothesis that the device traversed the myometrium before adhering to adjacent tissue.

Precise intraoperative localization can be challenging, particularly in cases of dense adhesion. Extrauterine migration of an IUD can pose a significant intraoperative challenge, particularly when the device is embedded within the omentum and obscured by adhesions. Current recommendations advocate surgical removal of extrauterine IUDs because of the potential risks of chronic inflammation, adhesion formation, visceral injury, and pelvic pain [7, 8]. Laparoscopy is widely accepted as the first‐line approach for both diagnosis and removal of an extrapelvic IUD migration in symptomatic patients, with reported success rates of approximately 64% [10]. Laparoscopy is considered the preferred approach due to its minimally invasive nature and favorable postoperative outcomes; however, successful retrieval may be hindered when the exact location of the device cannot be identified intraoperatively [2, 10]. In such situations, fluoroscopic guidance serves as a valuable adjunct by providing real‐time imaging and precise localization of the migrated device, thereby facilitating targeted dissection and reducing unnecessary tissue manipulation. Previous reports have demonstrated the utility of fluoroscopy in localizing occult extrauterine IUDs that were difficult to identify using conventional imaging or laparoscopy alone [11]. In the present case, the combination of fluoroscopic guidance and laparoscopy enabled accurate localization of an omentum‐embedded IUD, allowing safe retrieval through a minimally invasive approach while avoiding conversion to laparotomy. This strategy not only improved operative efficiency but also preserved the well‐established benefits of laparoscopy, including reduced postoperative pain, shorter hospital stay, faster recovery, and lower surgical morbidity. Therefore, fluoroscopy‐assisted laparoscopy may represent a useful option in selected cases of migrated IUDs with uncertain intra‐abdominal localization, particularly when dense adhesions or omental embedding obscure direct visualization. The patient reported being satisfied with the fluoroscopic assisted laparoscopy for the removal of the extrapelvic IUD migration. The procedure was perceived as efficient and minimally invasive, contributing to a positive overall experience. Additionally, the patient appreciated the rapid recovery following the surgery. The short postoperative period and quick return to normal activities further reinforced the patient′s satisfaction with the chosen treatment approach.

## 4. Conclusion

This case reinforces the clinical importance of maintaining early suspicion for extrapelvic IUD migration and applying a systematic imaging approach when symptoms or clinical findings are suggestive. When pelvic ultrasonography is inconclusive, adjunctive abdominal radiography with uterine markers can enhance diagnostic accuracy and guide further management. Consistent with this diagnostic strategy, the present case demonstrates that fluoroscopy‐assisted laparoscopy may serve as a valuable adjunct in selected cases of extrauterine IUD migration, particularly in the presence of dense adhesions or atypical device locations, enabling safer and more efficient removal while minimizing surgical morbidity.

## Author Contributions

Mohammad Haekal performed the surgery and reviewed the manuscript. Caroline Gladys Puspita assisted in the surgery and drafted the manuscript. Tiara Sirinding Mangiwa prepared the ethics documentation and managed journal submission. Mazkuroh Urfah contributed to manuscript drafting and journal submission. Gde Suardana supervised the surgery and critically reviewed the manuscript.

## Funding

No funding was received for this manuscript.

## Ethics Statement

Research ethics committees are responsible for ensuring the protection of the rights and welfare of human subjects that participate in clinical trials. All subjects gave their informed consent for inclusion before they participated in the study. The study was conducted in accordance with the Declaration of Helsinki, and the protocol was approved by the Ethics Committee of IRB/29/09/ETIK/2022.

## Conflicts of Interest

The authors declare no conflicts of interest.

## Supporting information


**Supporting Information** Additional supporting information can be found online in the Supporting Information section.

## Data Availability

The data that support the findings of this study are available on request from the corresponding author. The data are not publicly available due to privacy or ethical restrictions.
